# Multivariate effects of pH, salt, and Zn^2+^ ions on Aβ_40_ fibrillation

**DOI:** 10.1038/s42004-022-00786-1

**Published:** 2022-12-13

**Authors:** Hongzhi Wang, Jinming Wu, Rebecca Sternke-Hoffmann, Wenwei Zheng, Cecilia Mörman, Jinghui Luo

**Affiliations:** 1 Department of Biology and Chemistry, Paul Scherrer Institute, 5232 Villigen, Switzerland; 2 College of Integrative Sciences and Arts, Arizona State University, 85212 Mesa, AZ USA; 3 Department of Biosciences and Nutrition, Karolinska Institutet, 141 52 Huddinge, Sweden

**Keywords:** Protein aggregation, Biophysical chemistry, Peptides

## Abstract

Amyloid-β (Aβ) peptide aggregation plays a central role in the progress of Alzheimer’s disease (AD), of which Aβ-deposited extracellular amyloid plaques are a major hallmark. The brain micro-environmental variation in AD patients, like local acidification, increased ionic strength, or changed metal ion levels, cooperatively modulates the aggregation of the Aβ peptides. Here, we investigate the multivariate effects of varied pH, ionic strength and Zn^2+^ on Aβ_40_ fibrillation kinetics. Our results reveal that Aβ fibrillation kinetics are strongly affected by pH and ionic strength suggesting the importance of electrostatic interactions in regulating Aβ_40_ fibrillation. More interestingly, the presence of Zn^2+^ ions can further alter or even reserve the role of pH and ionic strength on the amyloid fibril kinetics, suggesting the importance of amino acids like Histidine that can interact with Zn^2+^ ions. Both pH and ionic strength regulate the secondary nucleation processes, however regardless of pH and Zn^2+^ ions, ionic strength can also modulate the morphology of Aβ_40_ aggregates. These multivariate effects in bulk solution provide insights into the correlation of pH-, ionic strength- or Zn^2+^ ions changes with amyloid deposits in AD brain and will deepen our understanding of the molecular pathology in the local brain microenvironment.

## Abstract (web summary)

Several essential brain micro-environments like pH, salt, and metal ions play a vital role in modulating the amyloid-ß fibrillation, however, the cooperative modulation of these variables remains poorly understood. Here, the authors investigate multivariate effects of pH, ionic strength, and Zn^2+^ ions on Aß_40_ peptide fibrillation.

## Introduction

Among neurodegenerative diseases, Alzheimer’s disease (AD) is the most prevalent one with a contribution of 60–70% to the global cases of dementia^1^. The disease is clinically manifested on the histological level by the deposition of amyloid-β (Aβ) senile plaques^2^ and tau neurofibrillary tangles^3^. The abnormal aggregation of the Aβ peptides and tau protein play an essential role in the development of AD. The most common isoforms of Aβ peptides are the Aβ40 and Aβ42 ones with a size of 4.3 kDa and 4.5 kDa, respectively. Aβ is negatively charged at physiological pH. The formation of Aβ fibrils occurs through the lag phase with forming transient and heterogeneous oligomers, and then through the elongation, and saturation phases with conversion into insoluble cross-β structures^4,5^. In many studies, the oligomers are found to be responsible for neuronal dysfunction through various toxic pathways, like synaptic dysregulation, membrane permeabilization and mitochondrial dysfunction. Though many anti-oligomer and anti-fibril approaches have been investigated^4,5^, so far no effective early diagnosis or therapy have been established. In addition to the oligomeric transiency and heterogeneity, intrinsically disordered Aβ is lack of a well-defined structure and is prone to interact with other constituents in the brain, complicating the aggregation pathway and adding serious challenges to the development of AD therapy. Several essential brain constituents, like pH, salt, and metal ions, play a vital role to modulate the secondary structural conversion, fibrillation kinetics, as well as toxicity of Aβ aggregates^5,6^.

In human AD brains, pH, salt, and metal ion concentrations differ from the healthy ones. The pH value ranges from 6.3 to 6.8 in human AD brains^7^, which is lower than that of the healthy ones with a range from 7.1 to 7.3^8^. A low pH is linked to brain acidosis and therefore inflammatory processes in AD. Extracellular and intracellular acidosis have been observed in the cerebrospinal fluid (CSF)^9^ and in the white matter of AD cases^10^, separately. Zn^2+^ ions increase up to 1 mM within amyloid plaques^11^, so does an ionic strength of sodium, potassium, or chloride ions in AD^12^. The concentration of Zn^2+^ ions is estimated to be much lower at about 150 μM in healthy human brain^13^. Compared to controls, sodium ions in frontal and parietal cortex regions of AD samples increase up to 25% and 20%, respectively^12^.

In vitro biophysical assays have been carried out to investigate how conditions such as pH, Zn^2+^ ions, or sodium ionic strength individually modulate the Aβ aggregation and toxicity^14,15^. The Aβ aggregation is highly influenced by the pH, which is facilitated during sample preparation where a high pH (app. pH 11) is used to solubilize the sample and to avoid aggregation before experiments are executed at physiological pH^16^. For the other extreme condition, a low pH down to 2 is used for formation of more homogenous samples of Aβ fibrils needed for structural analysis using cryo-EM^17^. The Aβ42 peptides are more prone to aggregation compared to Aβ40. For this reason, the Aβ42 peptides are commonly aggregated in vitro at pH 8 to slow down the aggregation to reach a suitable time window for aggregation kinetics experiments. The fibrillization rate below pH 7 decreases due to protonation of the Histidine residues in the N-terminal part of Aβ^18^. A lower pH prevents Aβ fibrillation, but in the range of 7–9, a higher pH only shows negligible inhibition against Aβ fibrillation^18^. It has also been observed that protonation of the Histidines stabilizes the assembly of Aβ fibrils at pH 6^19^. These reveal that decreasing pH slows down Aβ fibrillation and stabilizes the end-product fibril through the protonation under acidic conditions. In agreement with an increased ionic strength in human AD brains^12^, increased ionic strength by increasing concentration of sodium ions, accelerates the secondary nucleation rate in in vitro studies^15^, promotes Aβ40 fibrillation kinetics in a bulk solution^15,20^, and further modifies morphology of Aβ_40_ aggregates by shielding the Aβ electrostatic repulsion^15^. The Zn^2+^ ion is coordinated within the three Histidines (residues 6, 13, and 14) and weakly binds to residues 23 and 28 of the N-terminal Aβ^21^. At pH 7.2, sub-stoichiometric amounts of Zn^2+^ effectively retard Aβ_40_ fibrillation by reducing the elongation rate through the transient formation of the Aβ_40_-Zn^2+^ complex within the N-terminus^14,21^. At 25 µM, Zn^2+^ causes the rapid formation of congo red dye sensitive amyloid aggregates^22^. Briefly, Aβ is prone to different aggregation pathways even under slightly different conditions. It remains to be explored how these essential constituents cooperatively influence Aβ aggregation and morphology. The cooperative or multivariate effects of these constituents on amyloid aggregation will offer the comprehensive understanding towards the molecular basis of AD pathogenesis.

In this study, we implemented the protein crystallization robotics to create a series of the pH, salt, and Zn^2+^ constituents. The Aβ aggregation process is heterogeneous and is highly sensitive to slight variations of experimental conditions, but with this automation, highly accurate conditions can be prepared in addition to the possibilities of studying several factors simultaneously. The pH was varied from 6.5 to 8.0 and the salt concentration was varied from 0 to 0.1 M NaCl in the absence or presence of Zn^2+^ ions. With the multivariate conditions of pH, ionic strength, and Zn^2+^ ions, we investigated the cooperative effects of these essential constituents, on Aβ_40_ fibrillation kinetics as well as the morphologies of Aβ_40_ aggregates. The strength of this project was to study three different conditions simultaneously. The aim was to investigate how changes in pH and ionic strength modulate the Aβ_40_ aggregation, as well as how the Zn^2+^ ion modulation of the Aβ_40_ aggregation process is affected by a range of different pH and ionic strengths. With this approach, these observations of the cooperative effects enable us to carefully study amyloid fibrillation in vitro and correlate these constituent changes with the possible molecular pathogenesis in human AD brain.

## Results

### Buffer matrix design

To investigate how cooperative effects of multivariate conditions modulate the Aβ_40_ fibrillation process, a Thioflavin T (ThT) fluorescence assay was used to monitor the aggregation kinetics of 10 μM recombinant Aβ_40_ peptides. ThT is a commonly used fluorescence dye to monitor the formation of amyloid fibrils, as its fluorescence intensity sharply increases upon binding to amyloid fibrils^23^. The buffer matrix with multivariate conditions was programmed and dispensed with the protein crystallography FORMULATOR® screen builder. This liquid handler implements microfluidic technology and can dispense up to 34 different ingredients. The builder accurately generated a 96-condition buffer matrix shown in Table 1 with a series of pH values from 6.5 to 8.0 and NaCl concentrations from 0 M to 0.1 M in the presence or absence of 40 μM Zn^2+^ ions. For all conditions 20 mM sodium phosphate buffer was used. By conducting ThT fluorescence assays in a buffer matrix, we were able to observe the multivariate effects of two or three constitutes on Aβ_40_ fibrillation kinetics simultaneously (Figs. S1 and 1). The fibrillation kinetics assays in the buffer matrix have been repeated separately for four times with similar overall trends, and samples were tested in triplicates each time. The data from the experiments conducted for the first time are representable for the repeated measurements as well as the following analysis. The kinetic curves presented as average with standard error of the mean (SEM) are shown in Fig. S1.

**Table 1:** ThT buffers prepared with the FORMULATOR® - screen builder in a 96-deep well plate.

ThT buffers	20 mM potassium phosphate with 40 μM ThT in the absence of 40 μM Zn						20 mM potassium phosphate with 40 μM ThT in the presence of 40 μM Zn					
	1	2	3	4	5	6	7	8	9	10	11	12
A	pH 6.5 0 M NaCl	pH 6.8 0 M NaCl	pH 7.1 0 M NaCl	pH 7.4 0 M NaCl	pH 7.7 0 M NaCl	pH 8.0 0 M NaCl	pH 6.5 0 M NaCl	pH 6.8 0 M NaCl	pH 7.1 0 M NaCl	pH 7.4 0 M NaCl	pH 7.7 0 M NaCl	pH 8.0 0 M NaCl
B	pH 6.5 0.0143 M NaCl	pH 6.8 0.0143 M NaCl	pH 7.1 0.0143 M NaCl	pH 7.4 0.0143 M NaCl	pH 7.7 0.0143 M NaCl	pH 8.0 0.0143 M NaCl	pH 6.5 0.0143 M NaCl	pH 6.8 0.0143 M NaCl	pH 7.1 0.0143 M NaCl	pH 7.4 0.0143 M NaCl	pH 7.7 0.0143 M NaCl	pH 8.0 0.0143 M NaCl
C	pH 6.5 0.0286 M NaCl	pH 6.8 0.0286 M NaCl	pH 7.1 0.0286 M NaCl	pH 7.4 0.0286 M NaCl	pH 7.7 0.0286 M NaCl	pH 8.0 0.0286 M NaCl	pH 6.5 0.0286 M NaCl	pH 6.8 0.0286 M NaCl	pH 7.1 0.0286 M NaCl	pH 7.4 0.0286 M NaCl	pH 7.7 0.0286 M NaCl	pH 8.0 0.0286 M NaCl
D	pH 6.5 0.0429 M NaCl	pH 6.8 0.0429 M NaCl	pH 7.1 0.0429 M NaCl	pH 7.4 0.0429 M NaCl	pH 7.7 0.0429 M NaCl	pH 8.0 0.0429 M NaCl	pH 6.5 0.0429 M NaCl	pH 6.8 0.0429 M NaCl	pH 7.1 0.0429 M NaCl	pH 7.4 0.0429 M NaCl	pH 7.7 0.0429 M NaCl	pH 8.0 0.0429 M NaCl
E	pH 6.5 0.0571 M NaCl	pH 6.8 0.0571 M NaCl	pH 7.1 0.0571 M NaCl	pH 7.4 0.0571 M NaCl	pH 7.7 0.0571 M NaCl	pH 8.0 0.0571 M NaCl	pH 6.5 0.0571 M NaCl	pH 6.8 0.0571 M NaCl	pH 7.1 0.0571 M NaCl	pH 7.4 0.0571 M NaCl	pH 7.7 0.0571 M NaCl	pH 8.0 0.0571 M NaCl
F	pH 6.5 0.0714 M NaCl	pH 6.8 0.0714 M NaCl	pH 7.1 0.0714 M NaCl	pH 7.4 0.0714 M NaCl	pH 7.7 0.0714 M NaCl	pH 8.0 0.0714 M NaCl	pH 6.5 0.0714 M NaCl	pH 6.8 0.0714 M NaCl	pH 7.1 0.0714 M NaCl	pH 7.4 0.0714 M NaCl	pH 7.7 0.0714 M NaCl	pH 8.0 0.0714 M NaCl
G	pH 6.5 0.0857 M NaCl	pH 6.8 0.0857 M NaCl	pH 7.1 0.0857 M NaCl	pH 7.4 0.0857 M NaCl	pH 7.7 0.0857 M NaCl	pH 8.0 0.0857 M NaCl	pH 6.5 0.0857 M NaCl	pH 6.8 0.0857 M NaCl	pH 7.1 0.0857 M NaCl	pH 7.4 0.0857 M NaCl	pH 7.7 0.0857 M NaCl	pH 8.0 0.0857 M NaCl
H	pH 6.5 0.1 M NaCl	pH 6.8 0.1 M NaCl	pH 7.1 0.1 M NaCl	pH 7.4 0.1 M NaCl	pH 7.7 0.1 M NaCl	pH 8.0 0.1 M NaCl	pH 6.5 0.1 M NaCl	pH 6.8 0.1 M NaCl	pH 7.1 0.1 M NaCl	pH 7.4 0.1 M NaCl	pH 7.7 0.1 M NaCl	pH 8.0 0.1 M NaCl

**Fig. 1:**
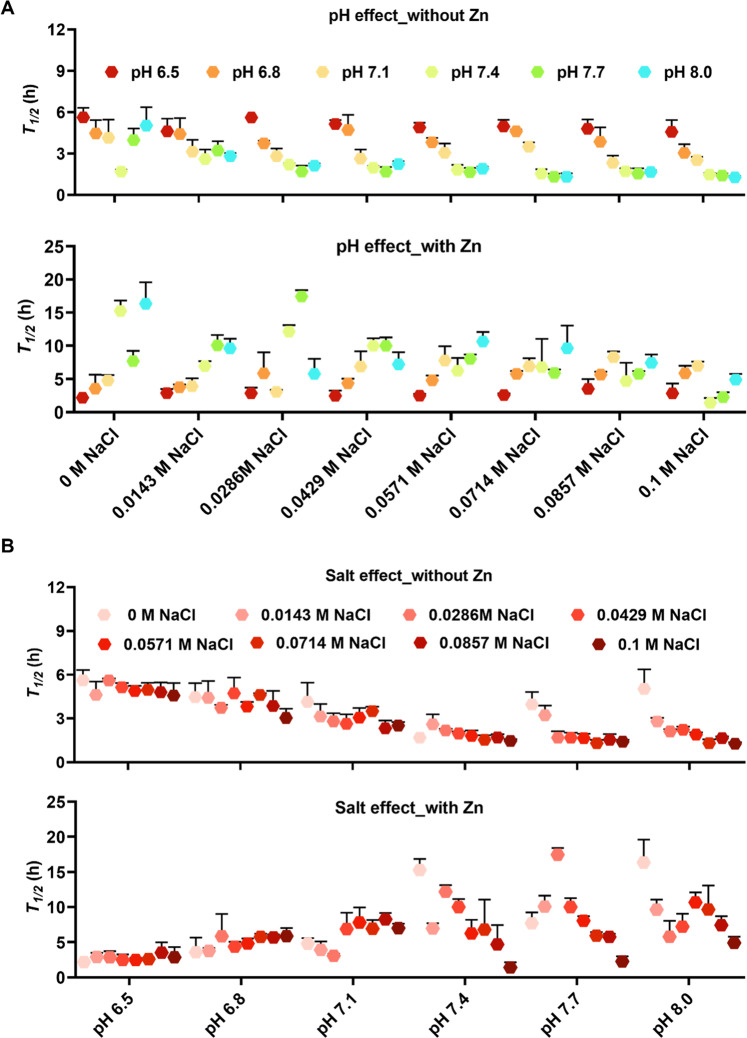
Multivariate effects of pH, ionic strength, and Zn^2+^ ions on Aβ_40_ fibrillation. The ${t}_{1/2}$ values of the fibrillation kinetics experiment of 10 µM Aβ_40_ in 20 mM potassium phosphate buffer at various pH values (6.5, 6.8, 7.1, 7.4, 7.7, and 8) and NaCl concentrations (0, 0.0143, 0.0285, 0.0429, 0.0571, 0.0714, 0.0857, and 0.1 M), were derived from sigmoidal curve fitting of ThT aggregation kinetics data of each repeat in the absence or presence of 40 µM Zn^2+^ ions. **A** pH effects and **B** salt effects on the Aβ_40_ fibrillation are shown separately for comparison (check text for details). All original ThT data were smoothed by choosing the Savitzky–Golay method with a points of Window from 5 to 30 using the Origin software before the aggregation halftime was extracted by sigmoidal curve fitting. The measurement variability is represented by error bars from the standard deviation of three replicas.

### Multivariate effects of pH, ionic strength, and Zn^2+^ ions on Aβ_40_ fibrillation

The combined effects of pH, ionic strength and metal binding on Aβ_40_ fibrillation were investigated with the varied NaCl concentrations ranging from 0 M to 0.1 M and the varied pH conditions from pH 6.5 to pH 8, in the absence or presence of 40 μM Zn^2+^ ions, by the ThT fluorescence kinetics assay in Fig. S1 and negative-staining transmission electron microscopy (TEM). Sigmoidal curve fitting of the aggregation kinetic traces from the ThT assay allowed us to extract the phenomenological parameter aggregation halftime (t_1/2_) for the different experimental conditions (Fig. 1A). At 0 M NaCl the aggregation kinetics were promoted, with a decrease in t_1/2_, by increasing the pH from 6.5 to physiological pH 7.4. Further increase of the pH towards pH 8 resulted in slower aggregation, in line with previous reports^18^. The effect of ionic strength was investigated by using a gradient of NaCl concentrations. Interestingly, the previous observed behavior was changed with an increase in ionic strength (see Fig. 1). In the presence of  increased NaCl concentrations, the aggregation kinetics were promoted, manifested by faster kinetics, for the whole pH range in a NaCl concentration-dependent manner. Clearly, the pH-dependent increase of t_1/2_ values from pH 7.4 to pH 8 for the 0 M NaCl condition was abolished in the presence of salt. Noteworthy, the highest effect of increasing ionic strength was at pH 8. In this study the salt effect was stronger for the higher pH values, whereas the presence of increasing salt concentration did not influence the aggregation kinetics significantly at pH 6.5. To conclude, ionic strength promotes Aβ_40_ fibrillation under all of the pH conditions studied and has a stronger impact at a higher pH than at physiological pH. Additionally, pH decrement from 7.4 to 6.5 prolongs the Aβ_40_ aggregation kinetics.

**Fig. 2:**
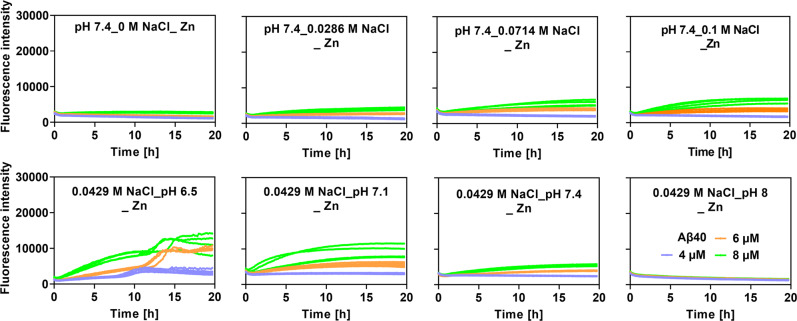
The multivariate effects of pH, salt, and Zn^2+^ ions on Aβ_40_ fibrillation at different Aβ_40_ concentrations (see legends). At 4 µM, Aβ_40_ displays fibrillation with flat curve in the presence of 40 µM Zn^2+^. All original ThT data were smoothed by choosing the Savitzky–Golay method with a points of Window from 5 to 30 using the Origin software.

The effects of pH and ionic strength were also investigated in the presence of Zn^2+^ ions. All conditions were measured in the presence of 40 μM Zn^2+^ ions and 10 μM Aβ peptides (Fig. S1). The Zn^2+^ ions concentration was relevant to the physiological conditions^13^. In Fig. 1A, pH decrement generally promotes Aβ fibrillation in the presence of Zn^2+^ ions. As pH decreases in the absence of Zn^2+^ ions, Aβ fibrillation suppresses. This may be explained by the protonation of Histidines. Overall, the aggregation kinetics were slower in the presence of Zn^2+^ ions at all NaCl concentrations and at pH above 6.5. In contrast, at pH 6.5 the aggregation kinetics was suppressed only at NaCl concentrations over 80 mM (Fig. 1B). At pH 6.5, the Histidine residues in the N-terminal part of Aβ are protonated and the Zn binding is weakened which may explain the observed results. Noteworthy, at pH above 7.0 in the presence of Zn^2+^ ions, the aggregation kinetics was clearly promoted with increasing ionic strength. This trend was also noted with varied Aβ concentrations at pH 7.4 (Fig. 2 top panel), while an increase in pH at a constant NaCl concentration exhibit an inhibitory effect of ThT activity (Fig. 2 bottom panel). Hence our data suggests that an increase in ionic strength promotes Aβ fibrillation above pH 7.0 in the presence of Zn^2+^ ions, in contrast to pH 6.5 where this effect is not as prominent for both in the presence and absence of Zn^2+^ ions.

To further understand how Zn^2+^ ions affect Aβ_40_ fibrillation at different Zn^2+^/Aβ_40_ ratios, we carried out the fibrillation at different concentrations of Aβ_40_ in the presence of NaCl. The fibrillation kinetics of Aβ_40_ at concentrations of 4, 6, or 8 µM, mainly displayed nonlinear curves at Zn^2+^/Aβ_40_ ratio of 5 or 10. Figure 2 further confirmed the effect of Zn^2+^ ions on the curves of Aβ_40_ fibrillation. In addition, Fig. 2 confirmed that under different conditions with varied combinations of micro-environmental constituents, only one constituent affect Aβ_40_ fibrillation completely, as shown in Fig. S1.

In summary, Aβ_40_ fibrillation behaves differently under various conditions, here studied with specific combinations of pH values, salt concentrations, and Zn^2+^ ions. Our results suggest that individual experimental conditions can be easily and accurately measured simultaneously taking multivariate factors into consideration.

### Electrostatic interactions bridge the impact of pH, ionic strength, and Zn^2+^ on Aβ_40_ conformations and aggregations

To further understand the underlying molecular mechanism governing the multivariate effect of these constituents on Aβ_40_ aggregation, we focused on the charged amino acids inside Aβ_40_, which could be affected by pH and ionic strength. Aβ_40_ has a typical sequence composition of a polyampholyte with an almost balanced composition of positively (3 Arg+Lys and 3 His) and negatively (6 Asp+Glu) charged amino acids. When varying the pH from 6 to 8, the only amino acid side chain with a pKa within that range is Histidine (pKa of ~6), which is expected to shift from partially protonated to deprotonated states. In addition, as suggested by a previous study that Zn^2+^ prefers to interact with the Histidine residues^24^ according to the Pearson acid base concept, the interplay between Zn^2+^, pH, and ionic strength close to physiological conditions can be nontrivial/important. We therefore performed molecular simulations capable of shedding light upon the interactions between the charged amino acids of Aβ_40_. Direct sampling of Aβ_40_ aggregation using molecular dynamics is challenging with the methods and computers available today. However, considering the reasonable amount of charged amino acids (12 out of 40) which could dominate the conformational preference of Aβ_40_ in the disordered state, we simulated the single-chain behavior of Aβ_40_ using a simple coarse-grained model to seek its correlation with aggregation.

**Fig. 3:**
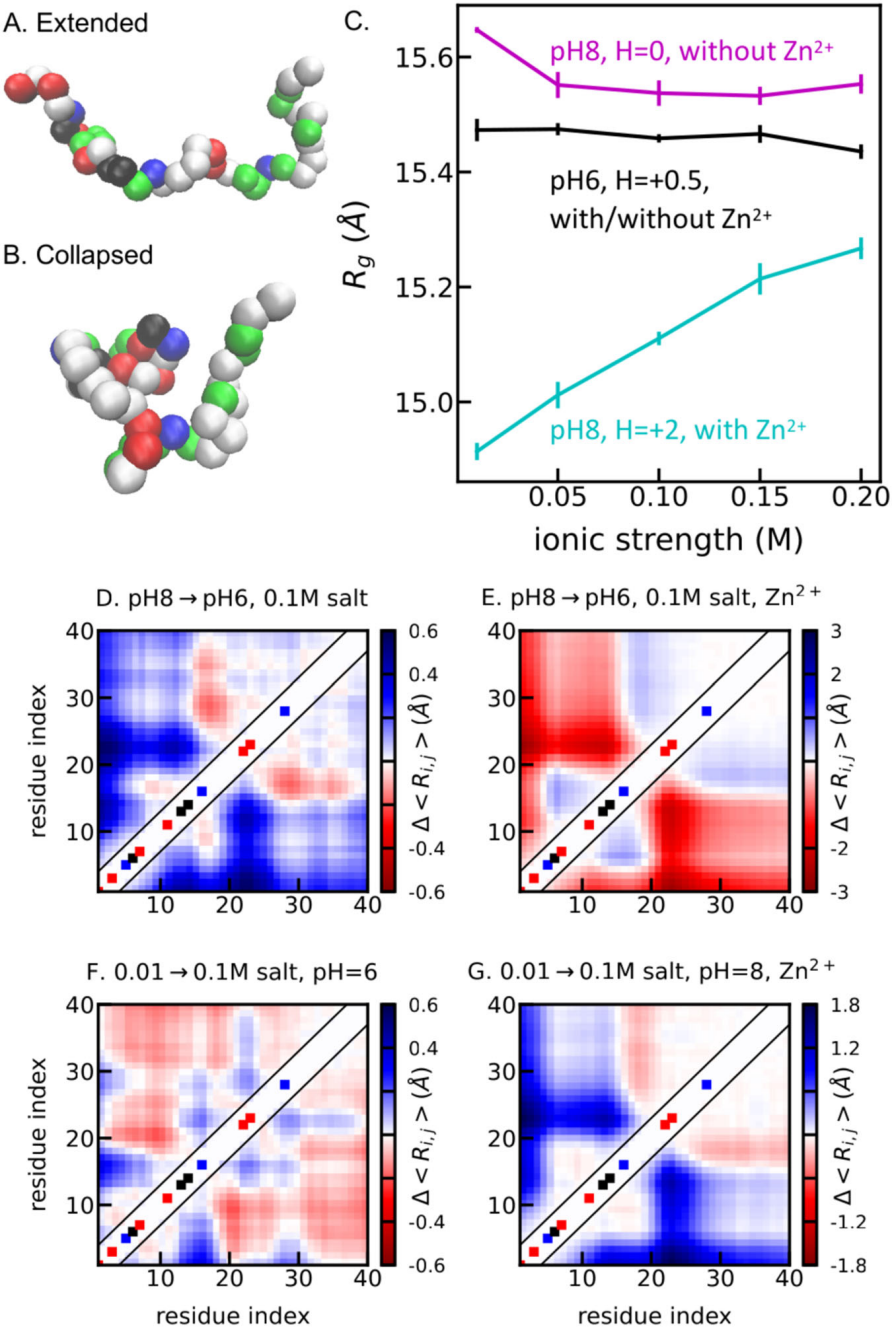
Size of Aβ_40_ from coarse-grained simulations at different ionic strength. **A**, **B** Two representative configurations with different radii of gyration (*R*_*g*_). Positively charged amino acids are highlighted in blue, negatively charged amino acids in red, Histidine in black, polar amino acids in green and nonpolar amino acids in white. **C** *R*_*g*_ from simulations for different conditions. Histidine is set to have different charges to model the effect of pH and Zn^2+^ ions: +0.5 for pH 6 with and without Zn^2+^ (black); 0 for pH 8 without Zn^2+^ (magenta); and +2 for pH = 8 with Zn^2+^ (cyan). **D** The difference of root mean squared distances (Δ*R*_*i,j*_) between simulations at pH 8 and 6 at the same ionic strength of 0.1 M without Zn^2+^ ions. Blue colors indicate larger distances between the i-th and j-th amino acids and red colors indicate smaller distances when increasing pH. **E** Δ*R*_*i,j*_ between pH = 8 and pH = 6 at the same ionic strength of 0.1 M with Zn^2+^ ions. **F** Δ*R*_*i,j*_ between ionic strengths of 0.1 M and 0.01 M at the same pH = 6 without Zn^2+^ ions. **G** Δ*R*_*i,j*_ between ionic strengths of 0.1 M and 0.01 M at the same pH 8 with Zn^2+^ ions. Colors in the diagonal of **D**–**G** show the positively (blue) and negatively (red) charged amino acids, and Histidine (black). The error bars of the simulation results are estimated using a block averaging method with five blocks.

We first explored the interplay between pH and Zn^2+^ ions in the simulations at different conditions. The physical variable used to characterize the conformation property is the radius of gyration (*R*_*g*_) capturing the size of Aβ_40_. A large *R*_*g*_ suggests extended conformations with more solvent exposed amino acids as illustrated in Fig. 3A in contrast to a small *R*_*g*_ suggesting collapsed conformations with buried amino acids in Fig. 3B. Noteworthy, for an IDP like the Aβ_40_ peptide with flexible conformations, Fig. 3A, B only illustrate representative conformations within a large pool of diverse conformations in the simulation. Without Zn^2+^ ions, the charge of Histidine is expected to vary from 0 to +0.5 with pH reducing from 8 to 6. As shown in Fig. 3C, *R*_*g*_ reduces when reducing the pH from 8 (magenta) to 6 (black). We further compared root means squared distances between every pair of amino acids (Δ*R*_*i,j*_) at the two different conditions in Fig. 3D. The N-terminal part of Aβ_40_ expands (blue) and the C-terminal part collapses (red) when reducing pH, whereas the size of the entire chain follows the N-terminal part due to the three Histidine and most charged amino acids located in this region.

When introducing Zn^2+^ ions, Zn^2+^ ions prefer to interact with Histidine at high pH and completely lose its interaction with Histidine at pH 5.5^24^. The simulation model with Zn^2+^ ions at pH = 6 is therefore the same as the one without Zn^2+^ ions at pH 6. We can then approximately capture the pH variation with Zn^2+^ ions by a simple model varying the charge of Histidine from +2 for pH 8 to +0.5 for pH 6. As shown in Fig. 3C, we saw a clear trend of collapsing when increasing the charge of Histidine from +0.5 (black) to +2 (cyan), which is due to the increasing attractive interactions inside the N-terminal part of the chain (Fig. 3E). Our simulation therefore suggests different pH dependences with and without Zn^2+^ ions, namely, when reducing pH the chain collapses without Zn^2+^ ions (Fig. 3D) and expands with Zn^2+^ ions (Fig. 3E). This correlates with the aggregation behaviors observed in the experiments (Figs. 1 and 2): pH decrement inhibits fibrillation without Zn^2+^ ions and promotes aggregation with Zn^2+^ ions. We can interpret the result as that a more extended conformation of Aβ_40_, in which amino acids are ready for intermolecular contacts, is present when varying Zn^2+^ and pH, which leads to self-assembly and aggregation.

We further investigated the interplay between salt, pH, and Zn^2+^ ions in the simulation. When increasing ionic strength at pH 6 without Zn^2+^ ions, we saw limited variation of the  Aβ_40_ size (black in Fig. 3C, F), whereas experiment suggested that salt weakly promotes Aβ_40_ aggregation at pH 6.5. Interestingly with Zn^2+^ ions, the experiments suggested the role of salt on aggregation shifts from weak inhibition to strong promotion when increasing pH from 6.5 to 8. This also correlates with the size of Aβ_40_ that we observed in the simulations, namely, Aβ_40_ expands at pH 8 with Zn^2+^ ions (cyan in Fig. 3C, G).

We note that for such a simple coarse-grained model, no other terms except the Coulombic interactions between charged amino acids are affected by salt, pH and Zn^2+^ ions. Therefore, for all the comparisons including varying both pH (Fig. 3D, E) and salt (Fig. 3F, G), the overall size of Aβ_40_ is mostly dependent on the N-terminal part of the chain where most of the charged amino acids and the three Histidine residues are located. We can then conclude that the interactions between the charged amino acids inside Aβ_40_ are sufficient to explain the multivariate effects of pH, ionic strength, and Zn^2+^ ions on Aβ_40_ aggregation.

### Multivariate effects of pH, ionic strength, and Zn^2+^ ions on the morphologies of Aβ_40_ aggregates

**Fig. 4:**
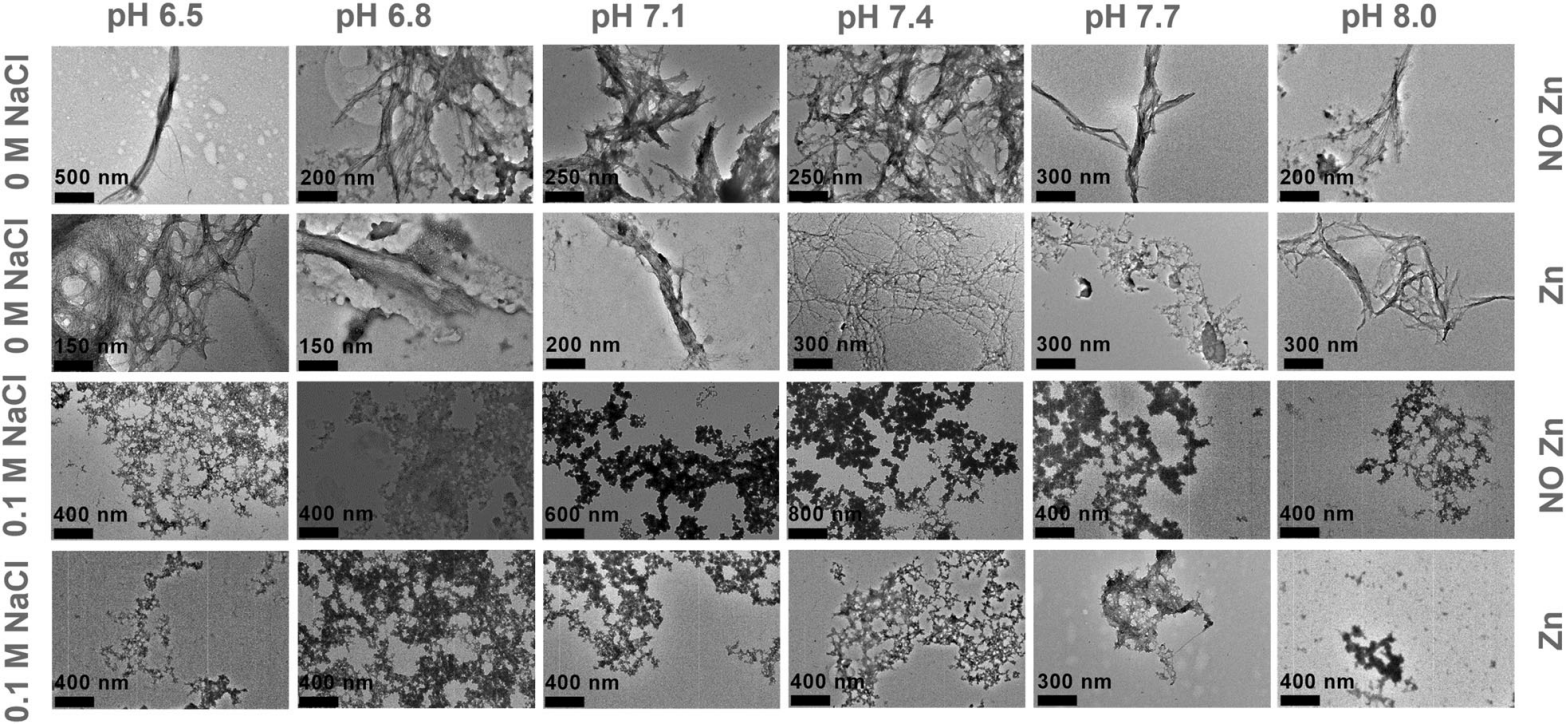
TEM characterization of Aβ_40_ aggregates. The samples of 10 μM Aβ_40_ in the presence or absence of NaCl and/or Zn^2+^ ions at different pHs were prepared after the incubation at 37 °C for 20 h without agitation.

To investigate the influence of pH, NaCl, and Zn^2+^ ions for the morphology of Aβ_40_ aggregates, 10 μM Aβ_40_ samples were taken from the ThT assay and were visualized using TEM. The TEM images shown in Fig. 4 reveal two different kinds of Aβ_40_ aggregates, fibrils and amorphous aggregates. In the absence of NaCl, Aβ_40_ peptide forms fibrils at all pH with and without Zn^2+^ ions. In the presence of 0.1 M NaCl, amorphous aggregates were observed independent of pH values and the presence of Zn^2+^ ions. This observation indicates that NaCl, rather than pH and Zn^2+^ ions, plays a role in changing the morphology of Aβ_40_ aggregates.

**Fig. 5:**
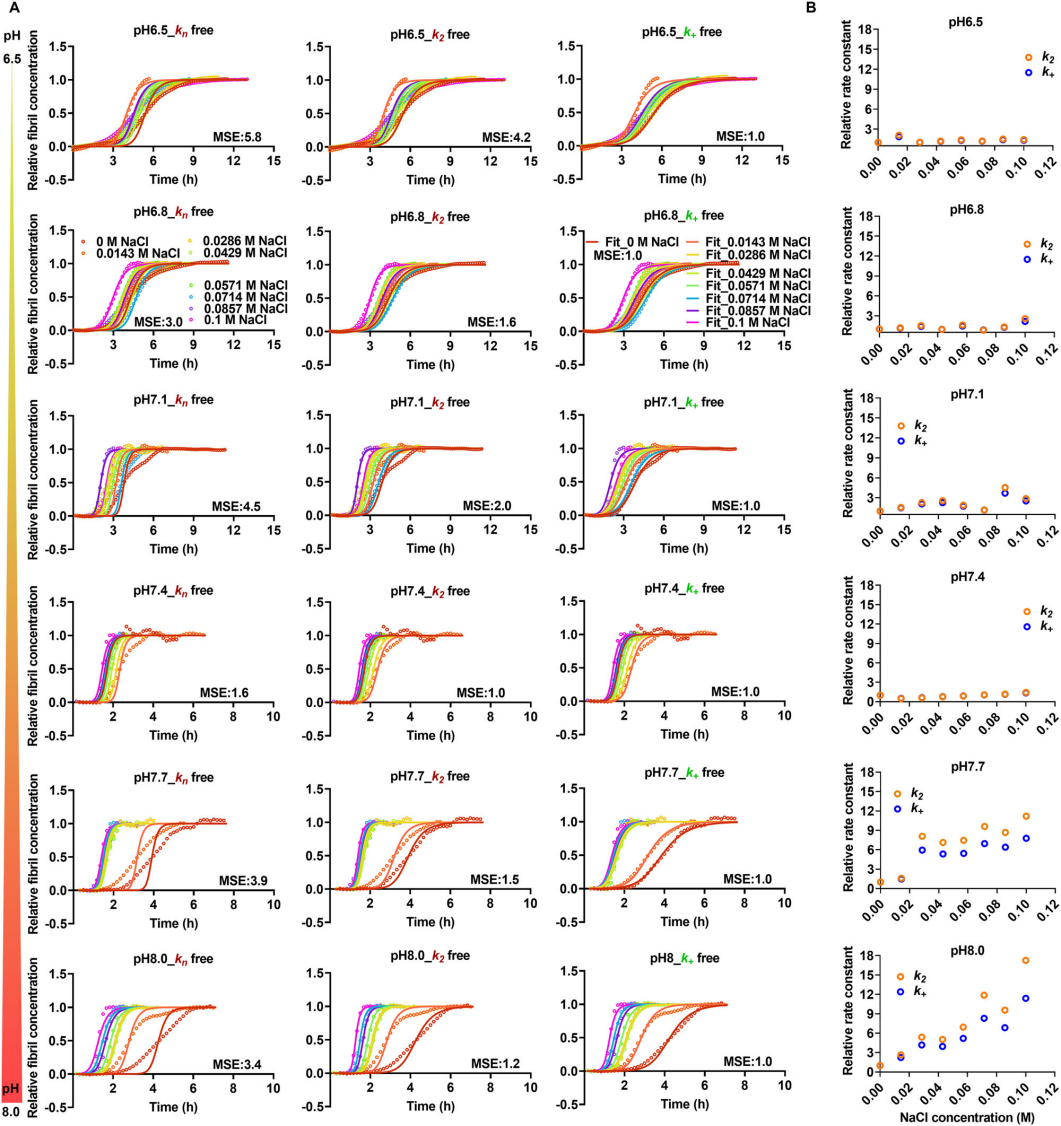
Different pH values and NaCl concentration mainly influence the secondary processes of the kinetics of Aβ_40_ fibrillation in the absence of Zn^2+^ ions. **A** Aggregation kinetics of 10 µM Aβ_40_ at different pH values (6.5, 6.8, 7.1, 7.4, 7.7, and 8) and concentrations of NaCl (0, 0,0143, 0.0285, 0.0429, 0.0571, 0.0714, 0.0857, and 0.1 M) were monitored by ThT fluorescence over time. The ThT data were then globally fitted by using the *AmyloFit* online software server^38^. For the fitting procedure, the data of Aβ_40_ at pH 7.4 in the absence of NaCl were first fitted with a secondary nucleation dominated model, from which a set of parameters including ${k}_{n}$ = 0.00047 in the unit of concentration^−nc+1^ time^−1^, ${k}_{2}$ = 4 × 10^7^ in concentration^−n2^ time^−1^, and ${k}_{+}$ = 9.49 × 10^8^ in concentration^−1^ time^−1^ of Aβ_40_ fibrillation were obtained and used as the initial guess values for the following global fit. Each one of the rate constants ${k}_{n}$, ${k}_{2}$, or ${k}_{+}$ was fitted freely, while the other two were set as initial guess values, by choosing the secondary nucleation dominated model. When ${k}_{+}$ and/or ${k}_{2}$, but not ${k}_{n}$, were freely fitted then the data was well described (see main text for details). The mean square error (MSE) values for each set of Aβ_40_ samples were normalized against the one with the best fit (lowest MSE value). **B** Relative rate constants (relative to the rate constants of Aβ_40_ at pH 6.5) derived from global fitting for Aβ_40_ samples at different pH values and salt concentrations.

To get further insight into the microscopic mechanisms of Aβ_40_ fibrillation at different pH values and NaCl concentrations, a global fit analysis of the ThT kinetic data in the absence of Zn^2+^ ions was conducted with an integrated rate law^25–28^ by using the AmyloFit online software server^29^. Amyloid proteins usually undergo aggregation via either primary or secondary dominated pathways^26,30^ and Aβ_40_ fibrillation is mainly dominated by secondary nucleation processes^30,31^. Therefore, we selected the secondary nucleation dominated model and first fitted the ThT data of Aβ_40_ at pH 7.4 in the absence of NaCl and Zn^2+^ ions before the global fit analysis (Fig. S2). A set of parameters, including the primary nucleation rate constant (${k}_{n}$) = 0.00047 in concentration^−nc+1^ time^−1^ (nc is the reaction order of primary nucleation that simply interprets a nucleus size), the secondary nucleation rate constant (${k}_{2}$) = 4e^+7^ in concentration^−n2^ time^−1^ (n_2_ is the reaction order of secondary nucleation), and the elongation rate constant (${k}_{+}$) = 9.49e^+8^ in concentration^−1^ time^−1^ of Aβ_40_ fibrillation process were obtained and used as the initial guess values for the following global fit analysis. Each one of the three rate constants (*k*_n_, *k*_2_, *k*_+_) was fitted freely, while the other two were kept. Then, the global fitting for all data with varied pH and NaCl concentrations was performed. The results of the global fit analysis shown in Figs. 5A and S3A indicate that if the rate constants ${k}_{2}$ and ${k}_{+}$,  rather than the ${k}_{n}$, were freely fitted, the fitting results can reproduce the curve shapes and the dependence of Aβ_40_ fibrillation on pH and NaCl, suggesting that the secondary pathways of Aβ_40_ aggregation are modulated by the multivariate experimental conditions. This observation regarding the impact of ionic strength is in line with previous reports^15^. In addition, the relative rate constants derived from the global fit shown in Figs. 5B and S3B are consistent with the aggregation half-times ${t}_{1/2}$ in Fig. 1. For instance, an increased relative rate constant in Figs. 5B and S3B matches with a decreased ${t}_{1/2}$ in Fig. 1, corresponding to the promotive effect of this specific pH value and concentration of NaCl on Aβ_40_ aggregation in Fig. S1. In summary, pH and salt modulate the fibrillation of Aβ_40_ peptide mainly via interfering with the secondary processes in the absence of Zn^2+^ ions. To shed more light to the mechanisms behind this observed behavior and to possibly distinguish between the ${k}_{2}$ and ${k}_{+}$ rate constants, seeding experiments were conducted.

### Seeding experiments

**Fig. 6:**
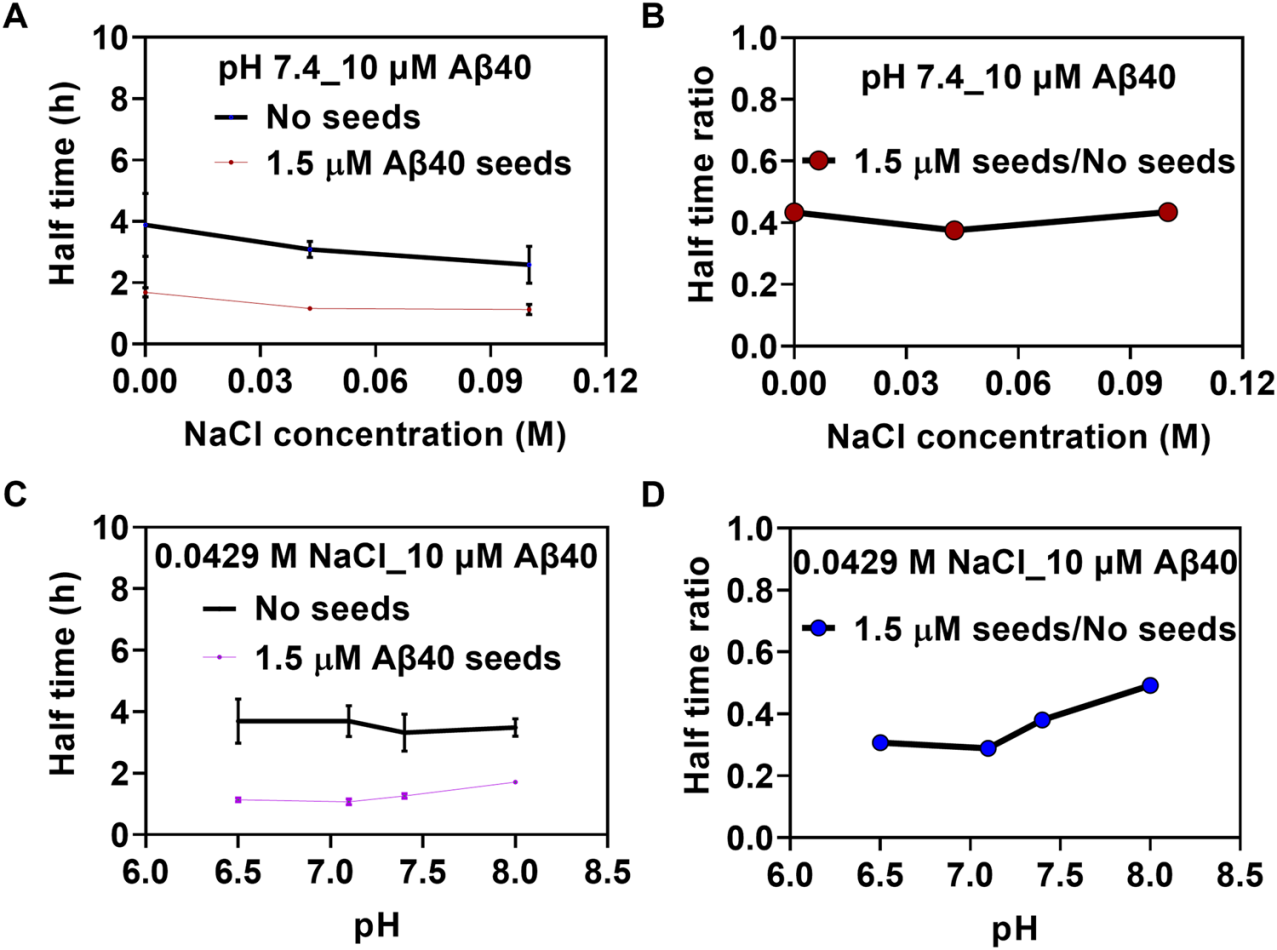
Seeding experiments of Aβ_40_ under the varied experimental conditions. The half time (t__1/2_) values of 10 μM Aβ_40_ kinetics at varied NaCl concentrations (**A**) and pH values (**C**) in the absence and presence of 1.5 μM Aβ_40_ seeds, shown as mean+SEM. The half time ratios (**B**) and (**D**) were obtained with averaged half times.

To investigate if the effects of pH and salt observed in the ThT kinetics data are dependent on the elongation or secondary nucleation processes of Aβ_40_ fibrillation, seeding experiments were performed. The original results (raw data) are shown in Fig. S4. In the presence of seeds, the contribution of primary nucleation is negligible compared to the secondary processes and hence the impact on *k*_+_ and *k*_2_ can be distinguished. 1.5 μM freshly prepared Aβ_40_ seeds were added to 10 μM monomeric Aβ_40_ peptides with three different NaCl concentrations at pH 7.4, as well as at four different pH with 42.9 mM NaCl. As expected, in the presence of seeds the aggregation kinetics were faster for all conditions where the plateau phase was reached before the elongation phase for unseeded conditions was started. Under these conditions the primary nucleation rate is low. This behavior indicates that the secondary nucleation processes are still the dominating mechanism in generating more fibril material. However, within the dataset with varied conditions of different pH and NaCl concentrations the presence of seeds did not change the kinetic traces significantly (Fig. 6A and C, which indicates that the secondary nucleation processes are the ones most likely affected during amyloid formation for alterations of both salt concentration and pH variations. Shown in Fig. 6B and D, we further calculated the half time ratio of Aβ_40_ fibrillation kinetics in the presence of these seeds to the absence of the seeds. At the varied NaCl concentrations and varied pH, the ratios remained similar. The half times of Aβ_40_ fibrillation kinetics in the absence or presence of 1.5 μM prepared Aβ_40_ seeds at varied NaCl concentrations and pH values were obtained via sigmoidal fitting and shown in Fig. 6A and C, respectively.

## Discussion

In this study, we prepared a buffer matrix including 96 different conditions with varied pH values and ionic strengths in the absence or presence of 40 μM Zn^2+^ ions using a protein crystallization screen builder. With the buffer matrix, we investigated the effect of multivariate conditions on Aβ_40_ fibrillation kinetics and the morphological changes of Aβ_40_ aggregates. We found that Aβ_40_ fibrillation can be affected through the interplay between pH, ionic strength and Zn^2+^ ions, summarized in Table 2. Decrement of pH from 8.0 to 6.5 possesses both promotive and inhibitory effects on Aβ_40_ fibrillation, depending on the presence of Zn^2+^ ions. In addition, the effect of pH on Aβ_40_ fibrillation can be further modulated by the concentration of NaCl. Increasing concentrations of NaCl salt generally promotes Aβ_40_ fibrillation at pH 7.4 and pH 8. Salt inhibits Aβ_40_ fibrillation at pH 6.5–7.1 in the presence of Zn^2+^ ions but promotes Aβ fibrillation in the absence of Zn^2+^ ions. Zn^2+^ ions slow down Aβ_40_ aggregation at both a lower and a higher pH in this study. These interplays may be achieved by regulating the secondary nucleation processes of Aβ_40_ fibrillation, as shown by the global fitting results in Figs. 5 and S3. Further, the morphology of Aβ_40_ aggregates changes in the presence of NaCl.

**Table 2:** Interplay between ionic strength, pH, and Zn^2+^ for Aβ fibrillation.

Zn^2+^	Impact of pH Increase	Impact of NaCl
Absence	Promote fibrillation	Promote fibrillation
Presence	Inhibit fibrillation	Inhibit fibrillation at pH 6
		Promote fibrillation at pH 8

The isoelectric point (pI) of Aβ_40_ is pH 5.4^32^ and the effective pI changes when interacting with specific ions^20^. The Histidines (H6/H13/H14) on the Aβ sequence have a pKa of 6.0^33^. In one study, the fibrillation rate of Aβ_42_ drops as pH decreases under pH 7, while the rate is independent of pH 7–9^18^. The concurrent protonation of H6/H13/H14 at low pH contributes to positive charges that repel each other, thereby stabilizing the peptides and preventing Aβ fibrillation^18^. The Histidine residues have been shown to be important for the fibrillation of Aβ^19^. Summarized in Table 2, our experimental and modeling studies support that in the absence of Zn^2+^ ions, the lower pH in the range from 7.4 to 6.5, a less yield of amyloid fibrils is obtained. Higher pH values (7.7 and 8.0) cannot significantly affect Aβ_40_ fibrillation compared to pH 7.4. An exception is the effect of pH on Aβ_40_ fibrillation in the absence of NaCl, where pH decrement from 8.0 to 7.4 promotes Aβ_40_ fibrillation. This promotive effect of pH decrease agrees with another study where reducing pH from 8.0 to 7.4 enhances the secondary nucleation of Aβ_42_ peptides, due to the attenuated electrostatic repulsion among Aβ_42_ peptides^34^. On the other hand, influences of primary nucleation events have also been reported by a recent study using different conditions with a range of pH values^35^. The present study further confirms that Aβ_40_ aggregation kinetics is extremely sensitive to relatively small changes in the experimental conditions, and provides a tool to prepare accurate buffer and multivariate conditions to limit such variations over a range of conditions such as pH values, salt-, and Zn ions concentrations. The aggregation of Aβ_42_ can be induced by the intra- and intermolecular salt bridges formed at pH 6–8, but not at pH < 5 and >9.0^36^. In addition, a rearrangement of the salt bridge network is involved in the misfolding of Apolipoprotein E4^37^. This corresponds to the pH effect on Aβ_40_ fibrillation in the absence of NaCl and Zn^2+^ ions that both pH decrease and increase from 7.4 inhibit Aβ_40_ fibrillation, presumably through the salt bridge change of D23 and K28. Zn^2+^ ions may compete with H^+^ ions at lower pH values (6.5, 6.8, and 7.1) for the H13 and H14 residues, thereby reducing their levels of protonation and leading to the decreased repulsion force among the Aβ_40_ peptides. Consequently, pH decrement from 7.1 to 6.5 promotes Aβ_40_ fibrillation compared with that at pH 7.1, in opposite of the effect of lower pH values in the absence of Zn^2+^ ions. These results are consistent with a previous study showing that a low pH of 6 has a strong stabilizing effect on Aβ fibrillation^19,38^. pH 7.4–8.0 in the presence of Zn^2+^ ions show irregular effects on Aβ_40_ fibrillation. This irregularity may be due to the integrated effect of pH on the protonation of side chains, salt on shielding electrostatic repulsion, and Zn^2+^ ions on binding to H6/H13/H14 of Aβ_40_ peptide. Of note, the Zn^2+^ ion concentration used in this study is higher compared to the peptide concentration, which may partially induce amorphous aggregates rather than amyloid fibrils that are detected in the ThT assay.

Ionic strength can shield the charge repulsion and may promote amyloid formation^6^. A previous study^15,20^ shows that salts can accelerate Aβ_40_ aggregation and modulate the morphological and structural changes of Aβ_40_ aggregates through electrostatic interactions, causing fibril polymorphism. Consistent with these studies, our results in the absence of Zn^2+^ ions indicate that increasing the concentration of NaCl from 0 M to 0.1 M promotes Aβ_40_ fibrillation (Figs. S1 and 1B) and the presence of 0.1 M NaCl converts Aβ_40_ fibrils into amorphous aggregates (Fig. 5). Salt can promote fibril formation, however, it can also inhibit fibril formation, leading to amorphous aggregates, depending on the concentration of salt^39^. These amorphous aggregates in the presence of 0.1 M NaCl may be reassembled from the fibril fragments through fibril fragmentation after the saturation of amyloid fibril formation. In addition, the promotive effect of NaCl on Aβ_40_ fibrillation is generally enhanced by pH increment from 6.5 to 8.0 and then the reduction of electrostatic repulsion at higher pH values. In the presence of Zn^2+^ ions, increasing salt concentrations prolongs Aβ_40_ fibrillation at pH 6.5–7.1, while promoting Aβ_40_ fibrillation at pH 7.4–8.0. The different effects of NaCl on Aβ_40_ fibrillation at low and high pH values may be explained by that Zn^2+^ ions complement with ionic strength differently for shielding the electrostatic repulsion among the Aβ_40_ peptides at different pHs. This effect remains to be further explored.

Zn^2+^ ions bind to the N-terminus (amino acids 1–16) of the Aβ peptides involving the H6/H13/H14 residues and D1 or sometimes E11 as the fourth binding ligand^40,41^. One study shows that under near-physiological conditions (pH 7.2–7.4), substoichiometric amounts of Zn^2+^ effectively retard Aβ_40_ fibrillation by reducing the elongation rate through the transient formation of the Aβ_40_-Zn^2+^ complex within the N-terminus^14^. In this study, Zn^2+^ ions at a final concentration of 40 μM possess opposite effects on Aβ_40_ fibrillation as pH values increase from 6.5 to 8.0. For instance, Zn^2+^ ions promote Aβ_40_ fibrillation at lower pH values like pH 6.5 and inhibit Aβ_40_ fibrillation at higher pH values like pH 8.0, compared with those at pH 7.4. This might be caused by the protonation of H6/H13/H14 at lower pH values (6.5, 6.8, and 7.1), leading to the reduction of Zn^2+^ ions binding to the H6/H13/H14 residues and consequently the decreased efficiency in inhibiting Aβ_40_ fibrillation. While higher pH values (7.7 and 8.0), the H6/H13/H14 residues may be further deprotonated compared to those at pH 7.4 and more Zn^2+^ ions can bind to H6/H13/H14, leading to the enhanced inhibitory effect of Zn^2+^ ions on Aβ_40_ fibrillation. In Aβ_40_ fibrillation, the Zn^2+^ effect varies at lower pH values as the concentration of NaCl increases from 0 M to 0.1 M. This can be explained that the Cl^-^ ions may counteract the charges of protonated H6/H13/H14 residues at a lower pH. However, Zn^2+^ ions have also been reported, under the physiological condition (pH 7.4), to rapidly induce the aggregation of the Aβ peptides in vitro^22^. The discrepancy compared with the above-mentioned studies could be explained by the different conditions used in different studies, as has been shown in this study that Zn^2+^ ions have different effects under various conditions.

Besides, our global fitting results indicate that pH and NaCl influence Aβ_40_ fibrillation by mainly interfering with the secondary nucleation process in the absence of Zn^2+^ ions (Figs. 5 and S3), which is in line with a previous study^15^. The addition of salt may shield the charge repulsion between the ends of the existing fibrils and free Aβ_40_ monomers that are about to be added to the fibril ends.

The micro-environmental constituents, like pH, salt, and metal ions, change in the brain during the progression of AD^7,8,11,12,42^. This change can modulate the abnormal aggregation of the Aβ_40_ peptides^5,6^. Though the effects of pH, salt, and Zn^2+^ ions on Aβ_40_ aggregation have been individually investigated, neither has the consensus been reached^14,18,19,22^, nor have the multivariate effects been studied. However, Aβ_40_ fibrillation kinetics is prone to alteration even with minor micro-environmental change. In this study, although we have not yet investigated the pH effect in the whole range, pH 6.5–8 substantially covers the micro-environmental changes in the AD brain. Low pH values used in vitro can mimic the acidosis, which is usually linked to inflammatory processes in vivo. The multivariate effects of pH, ionic strength, and Zn^2+^ ions on Aβ_40_ fibrillation may clarify the discrepancy in this field and deepen our understanding of the molecular pathogenesis of AD.

## Materials and methods

### Materials and sample preparation

Recombinant Aβ_40_ peptides were purchased from AlexoTech and the stock solutions were prepared by dissolving the lyophilized powder in 10 mM NaOH to a concentration of 2 mg/mL and then sonicated in an ice-water bath for 1 min, and filtered with a 0.2 μm centrifugal filter unit at 4 °C. All other agents, including potassium phosphate dibasic and potassium phosphate monobasic stocks, were purchased from Sigma-Aldrich. ThT stock solution was prepared to 3 mM in Milli-Q water. Zinc chloride and sodium chloride stock solutions were prepared by dissolving the metal salt in Milli-Q water to concentrations of 1 M and 5 M, respectively. All of buffers and stock solutions including 10 mM NaOH were filtered with 0.2 μm syringe-driven filters.

### ThT buffer preparation with FORMULATOR®

To prepare buffers used in ThT assays, potassium phosphate dibasic and potassium phosphate monobasic stocks were mixed at two different volume ratios, yielding potassium phosphate stocks at final concentrations of 1 M and pH values of 6 and 8, respectively. 1 mM zinc chloride was prepared by diluting the 1 M stock solution with Milli-Q water. ThT buffers were then prepared with the FORMULATOR® by dispensing the potassium phosphate stocks at pH 6 and pH 8 at 6 volume ratios, 8 different volumes of 5 M sodium chloride stock solution, and 3 mM ThT stock and 1 mM zinc chloride at constant volumes. The yielded ThT buffers (20 mM potassium phosphate) contained ThT at a final concentration of 40 μM in the absence or presence of zinc chloride (40 μM), while pH values change from pH 6.5 to pH 8 along the columns and sodium concentrations vary from 0 M to 0.1 M along the rows in a plate with 96 deep wells. The detailed information on the ThT buffers is shown in Table 1.

### ThT fluorescence assays

To study the effects of pH, salt (NaCl) and Zn^2+^ ions on the fibrillation kinetics of Aβ_40_ peptide, ThT assays were conducted immediately after the buffers were prepared with the FORMULATOR®. Samples were prepared by dispensing Aβ_40_ stock solution into the wells of a transparent 96-well plate manually, and then mixing thoroughly with the freshly prepared ThT buffers with a multiple channel pipette, yielding Aβ_40_ samples at a final concentration of 10 μM in 96 different ThT buffers as described in Table 1. In all, 30 μL of each sample was then transferred from the 96-well plate into a 384-well, non-treated black plate with transparent bottom (NUNC) and sealed with a piece of foil film. All samples were prepared in triplicate on ice. The 384-well plate was incubated in a microplate reader (PHERAstar FSX, BMG LABTECH, Germany) and the fluorescence kinetics of Aβ_40_ was monitored at 37 °C without agitation every 5 min, using wavelengths of 430 nm and 480 nm for excitation and emission, respectively.

All of the original ThT data were smoothed by choosing the Savitzky-Golay method with a Points of Window from 5 to 30 using Origin (Version 2018, OriginLab, USA). The smoothed data were then plotted with Prism (Version 8.0, GraphPad Software), as shown in Fig. S1.

To investigate the effect of Zn^2+^ ions on the Aβ_40_ aggregation at different pH and NaCl concentration, ThT experiments were also conducted at Aβ_40_ concentrations of 4, 6, and 8 μM (at NaCl concentrations of 0, 0.0286, 0.0714, and 0.1 M, pH7.4, **or** pH 6.5, pH 7.1, pH7.4, and pH 8, NaCl concentration of 0.0429 M), in the absence or presence of 40 μM Zn^2+^ ions, under same conditions mentioned above.

For seeding experiments, 10 μM Aβ_40_ seeds were prepared in 20 mM potassium phosphate buffer, pH 7.4, under same conditions mentioned above, incubate until the early plateau phase of Aβ_40_ aggregation, followed by sonication in ice-water bath for 2 min. Seeding experiments were performed with 10 μM Aβ_40_ at NaCl concentrations of 0, 0.0714, and 0.1 M, pH 7.4, or pH 6.5, pH 7.1, pH 7.4, and pH 8, NaCl concentration of 0.0429 M, in the absence or presence of 1.5 μM Aβ_40_ seeds, with same method in Fig. S1.

Original data were plotted with Prism (Version 8.0, GraphPad Software).

### Sigmodial fitting

To estimate the half time ${t}_{1/2}$ of Aβ_40_ aggregation kinetics, the sigmoidal fitting of individual curves was performed with smoothed data by using Eq. (1) with Origin (Version 2018, OriginLab, USA).

1 $$y=\frac{{y}_{{baseline}}{-y}_{{plateau}}}{1+{e}^{(t-{t}_{0})/{dt}}}+{y}_{{plateau}}$$

where ${{y}}_{{baseline}}$ and ${y}_{{plateau}}$ are the values of the data at the baseline and the plateau,$\,t$ is the time of amyloid aggregation course and ${t}_{0}$ is the time when the fluorescence intensity reaches half of the plateau value, while ${dt}$ is the time constant. And $y$ is the fitted value of the data at time $t$. The values of ${{y}}_{{baseline}}$ and ${y}_{{plateau}}$ were initially determined automatically by Boltzmann function. When parameters were set before fitting, ‘fixed’ options corresponding to ${{y}}_{{baseline}}$ and ${y}_{{plateau}}$ were left uncrossed, except those corresponding to ${{y}}_{{baseline}}$ of some curves obtained in the presence of 40 μM Zn^2+^ ions at pH 6.5, pH 6.8, pH 7.1, or pH 7.4, which were crossed. ${t}_{1/2}$ of aggregation kinetics were given by Eq. (2) and plotted with Prism (Version 8.0, GraphPad Software).

2 $${t}_{1/2}={t}_{0}$$

### Global fitting

To identify how pH and salt affect the microscopic rate processes of Aβ_40_ aggregation, the averaged ThT data obtained in the absence of Zn^2+^ ions were smoothed with the same method used for the smooth process of individual curves and fitted globally with an integrated rate law^25,26^ in AmyloFit online software server^29^ by using the method in our previous study^16^. Briefly, the secondary nucleation dominated model was selected, the data of Aβ_40_ at pH 7.4 in the absence of NaCl was first fitted, obtaining a set of parameters, which were used as the initial guess values for the following fits. Among these obtained parameters, the primary nucleation rate constant ${k}_{n},$ secondary nucleation rate constant ${k}_{2}$, or the elongation rate constant ${k}_{+}$ was fitted freely while the other two rate constants were set as fixed initial values. For detailed definitions of these parameters and fitting procedure, please refer to the nature protocol^29^ and our previous study^16^. The fitting results are shown in Figs. 3 and S3.

### Transmission electron microscopy

For TEM assay, the Formvar-coated, carbon-stabilized copper grids (400 mesh, from Ted Pella Inc., Redding CA) were glow-discharged (20 mA for 20 s). 10 μM Aβ_40_ samples were taken from ThT assays conducted at different pH values in the absence or presence of Zn^2+^ ions and/or NaCl. 4 μl of each sample was loaded on the discharged grid and incubated for 30 s, the excess samples on the grids were blotted with a piece of filter paper. 3.5 μl of 2% uranyl acetate was immediately added onto the grid and the excess stain solution was blotted after incubation for 30 s. The staining process was performed twice. The grids were then washed with 6 μl of Milli-Q water and air-dried. The negatively stained samples were imaged on a transmission electron microscope (PSI, Switzerland) operating with an accelerator voltage of 80 kV.

### Molecular dynamics simulations

We started with a residue-based coarse-grained model, HPS model^43^, which was parameterized for studying liquid-liquid phase separation (LLPS) of intrinsically disordered proteins (IDPs)^44,45^. In the original model, each amino acid was represented by a bead with charge (+1, 0, −1) and hydropathy^46^. There were three types of interactions: bonded interactions, electrostatic interactions, and short-range pairwise interactions. The bonded interactions were characterized by a harmonic potential with a spring constant of 10 kJ/Å^2^ and a bond length of 3.8 Å. The electrostatic interactions were modeled using a Coulombic term with Debye-Hückel electrostatic screening^47^ to account for the salt concentration. The short-range pairwise potential accounted for both protein-protein and protein-solvent interactions with an adjustable parameter $\epsilon$ for the interaction strength, which can be optimized using the experimental size of Aβ_40_. We further added additional terms for angle and dihedral preferences: a statistical angle potential from a previous study^48^ for all types of amino acids and a statistical dihedral potential published previously^49^. We found that an $\epsilon$ of 0.13 kcal/mol best captured the experimental Förster Resonance Energy Transfer (FRET) measurement of Aβ_40_^50^. All simulations were run at 298 K maintained by a Langevin thermostat with a friction coefficient of 0.01 ps^−1^ using HOOMD-Blue v2.9.2^51^. For each condition (salt or pH), the simulation was run for 2 μs with the first 100 ns dumped for equilibration of the system before data collection. The error bars were calculated using a block averaging method with five blocks.

## Supplementary information

Supplementary information

## Data Availability

The data presented in the figures of this article are available from the corresponding author upon reasonable request.
