# Supplementary material for: Multivariate effects of pH, salt, and Zn2+ ions on Aβ40 fibrillation
Source: Commun Chem. 2022 Dec 13;5:171. doi: 10.1038/s42004-022-00786-1 (PMC9814776; doi:10.1038/s42004-022-00786-1)
Supplement: Supplementary file 1 — Supplementary information [file 42004_2022_786_MOESM1_ESM.pdf]

Supplementary information

Multivariate effects of pH, salt, and Zn^{2+} ions on $A\beta_{40}$ fibrillation

Hongzhi Wang[1], Jinming Wu[1], Rebecca Sternke-Hoffmann[1], Wenwei Zheng[2], Cecilia Mörman[1,3], Jinghui Luo[1*]

1. Department of Biology and Chemistry, Paul Scherrer Institute, 5232 Villigen, Switzerland.
2. College of Integrative Sciences and Arts, Arizona State University, AZ 85212, Mesa, USA.
3. Department of Biosciences and Nutrition, Karolinska Institutet, 141 52 Huddinge, Sweden.

*Corresponding author: Jinghui.luo@psi.ch, Tel: +41 56 310 47 64

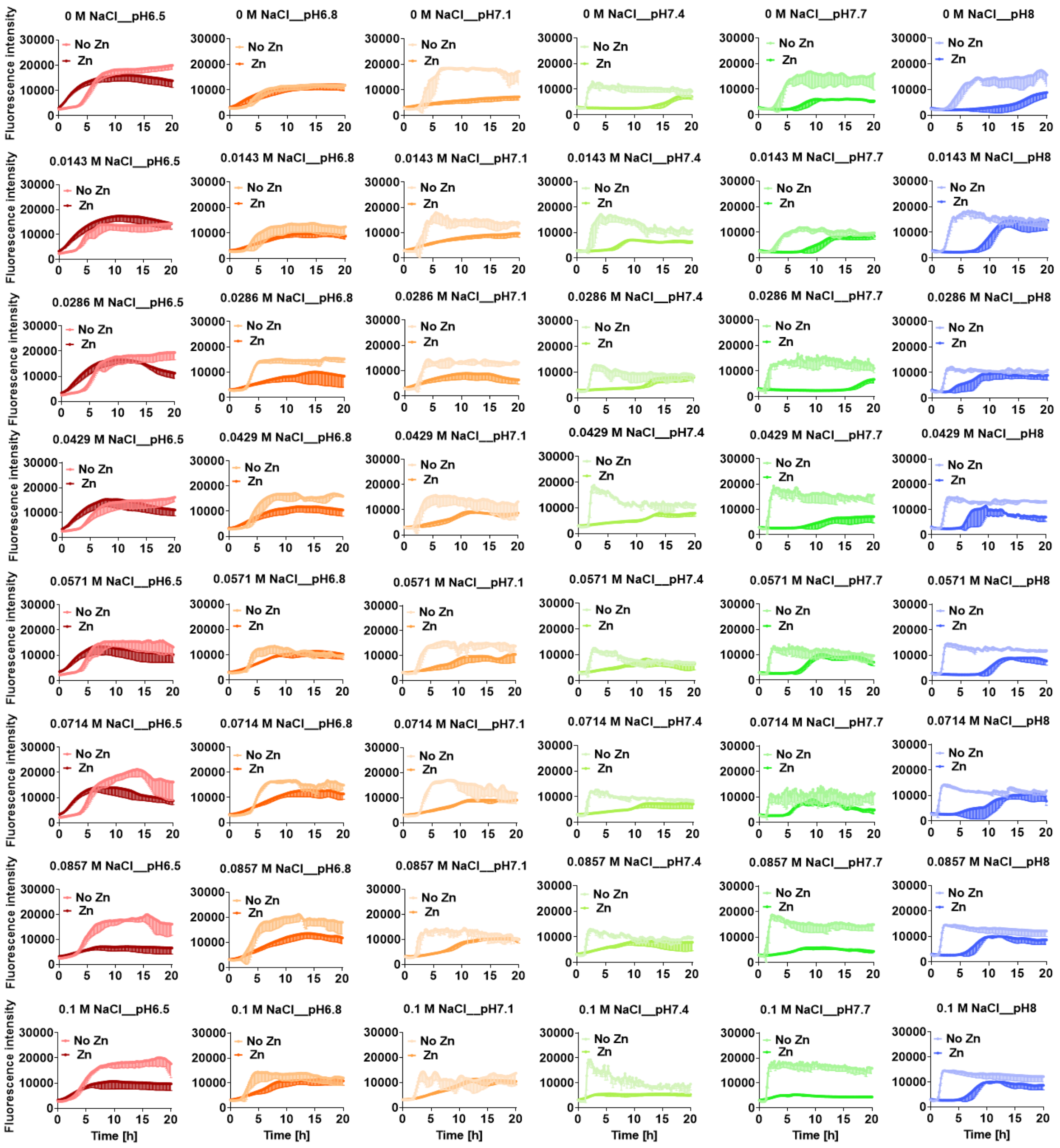

Supplementary Figure 1. Effects of pH, NaCl, and Zn^{2+} ions on $A\beta_{40}$ fibrillation. Aggregation kinetics of 10 μM $A\beta_{40}$ were monitored by ThT assays at 37°C under quiescent conditions. The results indicate that effects of pH, salt, and Zn^{2+} ions on $A\beta_{40}$ aggregation are influenced mutually. All original ThT data were smoothed by choosing the Savitzky-Golay method with a points of Window from 5 to 30 using the Origin software. The measurement variability is represented by error bars from standard error of the mean (SEM) of 3 replicas.

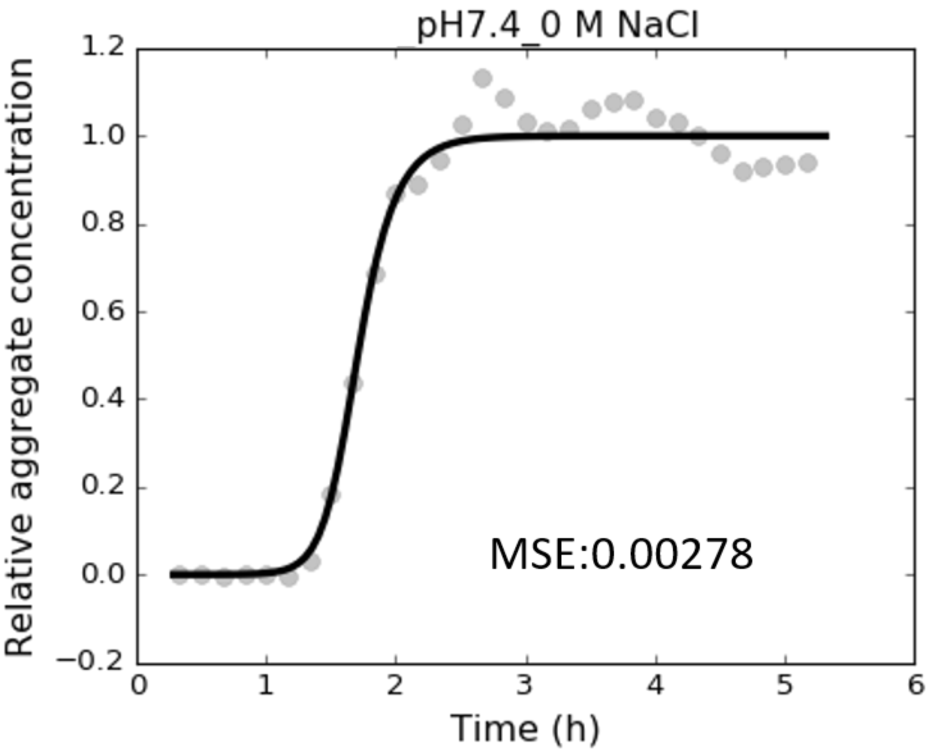

Supplementary Figure 2. Global fitting analysis of ThT data from 10 μM Aβ$_{40}$ alone at pH 7.4, in the absence of NaCl and Zn^{2+} ions. Aβ$_{40}$ fibrillation is a secondary nucleation dominated process. Following this fit, a set of parameters were obtained: k_n = 0.00047 in concentration^{-nc+1} time^{-1}; k_2 = 4e^{+7} in concentration^{-n2} time^{-1}; k_+ = 9.49e^{+8} in concentration^{-1} time^{-1}, which were uses as the initial guess values for the following fitting of Aβ$_{40}$ at varied NaCl concentrations and pH values, in the absence or presence of Zn $^{2+}$.

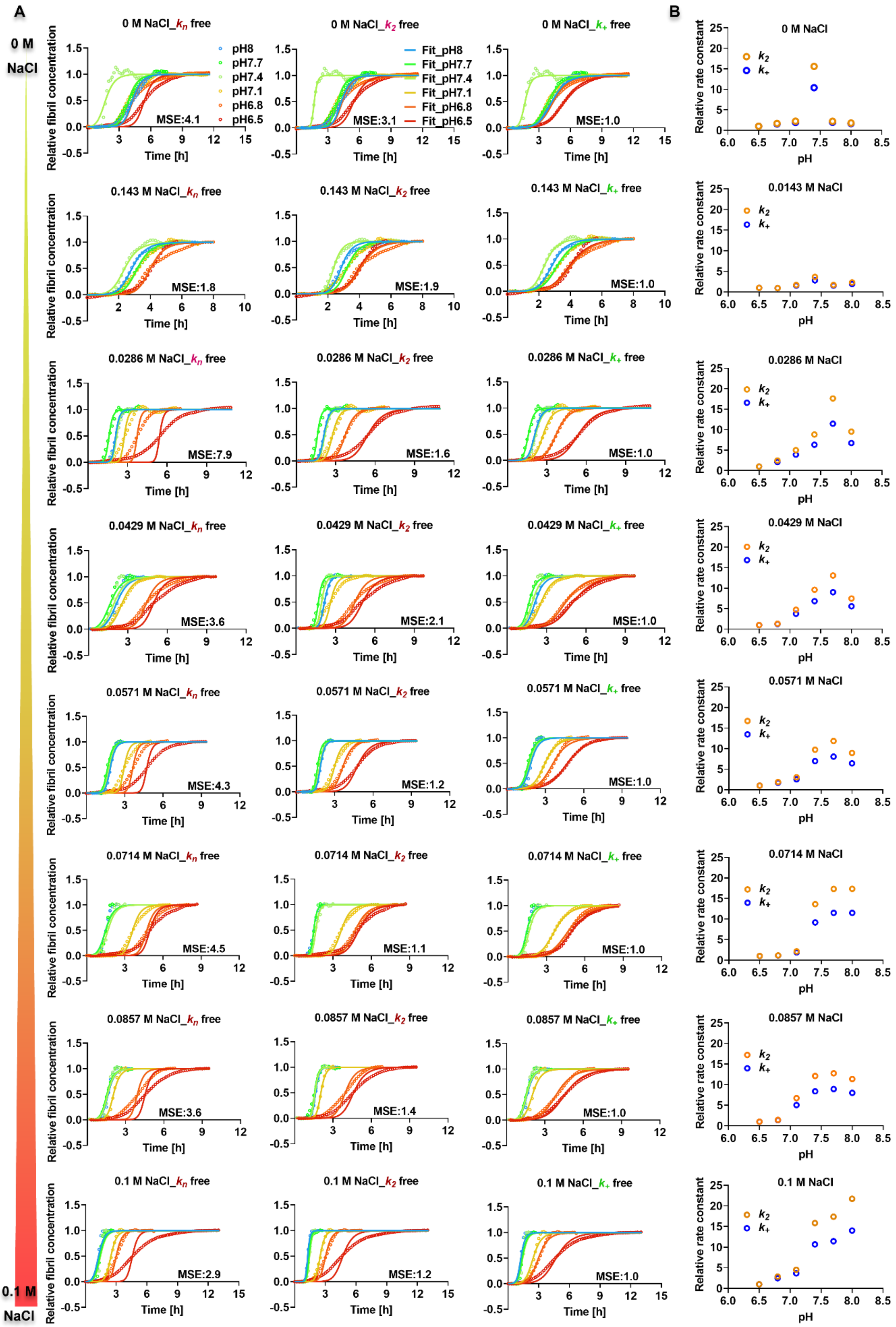

A
B
0 M NaCl
0.1 M NaCl
0 M NaCl_kn free
0 M NaCl_k2 free
0 M NaCl_k+ free
pH8
pH7.7
pH7.4
pH7.1
pH6.8
pH6.5
Fit_pH8
Fit_pH7.7
Fit_pH7.4
Fit_pH7.1
Fit_pH6.8
Fit_pH6.5
MSE:4.1
MSE:3.1
MSE:1.0
0.143 M NaCl_kn free
0.143 M NaCl_k2 free
0.143 M NaCl_k+ free
MSE:1.8
MSE:1.9
MSE:1.0
0.0286 M NaCl_kn free
0.0286 M NaCl_k2 free
0.0286 M NaCl_k+ free
MSE:7.9
MSE:1.6
MSE:1.0
0.0429 M NaCl_kn free
0.0429 M NaCl_k2 free
0.0429 M NaCl_k+ free
MSE:3.6
MSE:2.1
MSE:1.0
0.0571 M NaCl_kn free
0.0571 M NaCl_k2 free
0.0571 M NaCl_k+ free
MSE:4.3
MSE:1.2
MSE:1.0
0.0714 M NaCl_kn free
0.0714 M NaCl_k2 free
0.0714 M NaCl_k+ free
MSE:4.5
MSE:1.1
MSE:1.0
0.0857 M NaCl_kn free
0.0857 M NaCl_k2 free
0.0857 M NaCl_k+ free
MSE:3.6
MSE:1.4
MSE:1.0
0.1 M NaCl_kn free
0.1 M NaCl_k2 free
0.1 M NaCl_k+ free
MSE:2.9
MSE:1.2
MSE:1.0
Relative fibril concentration
Time [h]
0 M NaCl
0.0143 M NaCl
0.0286 M NaCl
0.0429 M NaCl
0.0571 M NaCl
0.0714 M NaCl
0.0857 M NaCl
0.1 M NaCl
k2
k+
Relative rate constant
pH

Supplementary Figure 3. Salts mainly influence the secondary processes of Aβ40 fibrillation in the absence of Zn^{2+} ions. (A) Aggregation kinetics of 10 µM Aβ40 at different pH values (6.5, 6.8, 7.1, 7.4, 7.7, and 8) and NaCl concentrations (0, 0,0143, 0.0285, 0.0429, 0.0571, 0.0714, 0.0857, and 0.1 M) were monitored by ThT fluorescence over time (the raw data can be found in **Fig.1**). The ThT data were then globally fitted with the same method used in **Fig.3** in the main manuscript by using the *AmyloFit* online software server [38]. ❖ The mean square error (MSE) values for each set of Aβ40 samples were normalized against the one with the best fit (lowest MSE value). **(B)** Relative rate constants (relative to the rate constants of Aβ40 at 0 M NaCl) derived from global fitting for Aβ40 samples at different pH values and salt concentrations. All original ThT data were smoothed by choosing the Savitzky-Golay method with a points of Window from 5 to 30 using the Origin software.

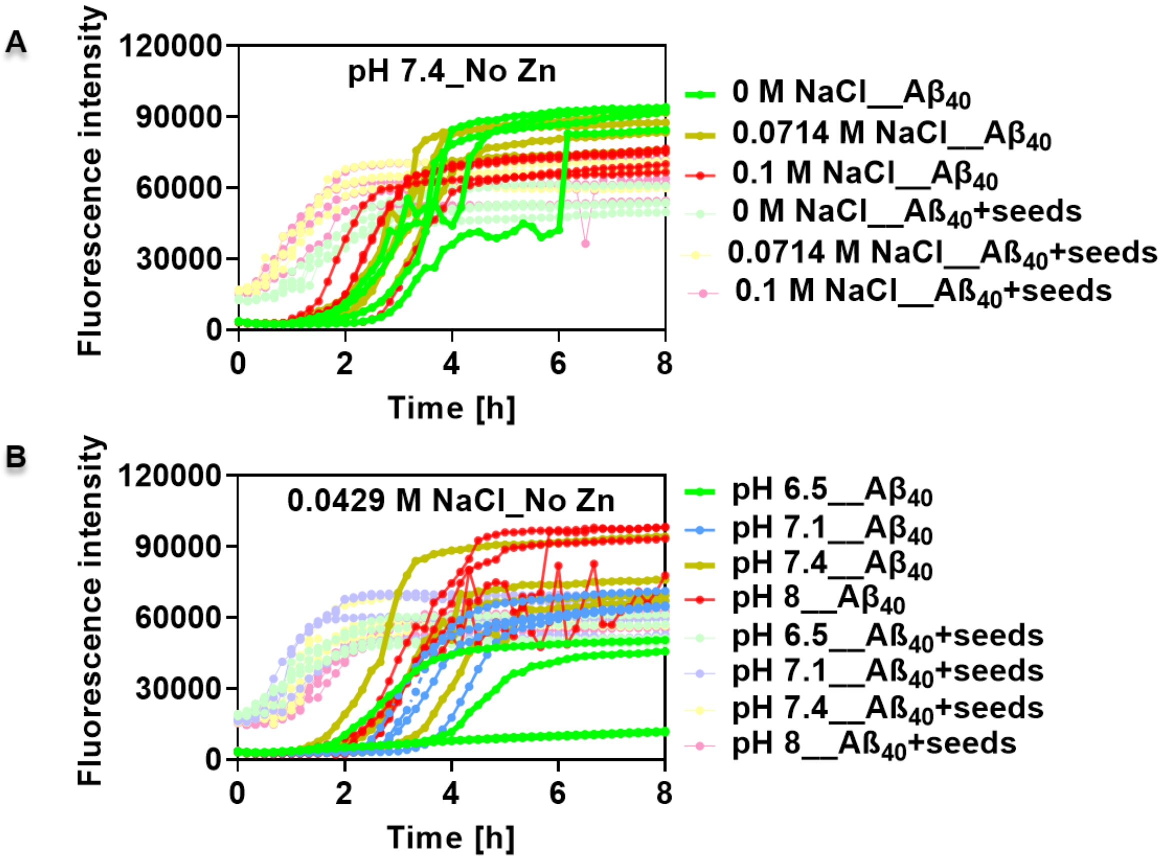

Supplementary Figure 4. Seeding experiments. The ThT kinetic experiments were performed with 10 µM Aβ40 in 20 mM potassium phosphate buffer at NaCl concentrations of 0, 0.0714, and 0.1 M, pH 7.4, or pH 6.5, pH 7.1, pH 7.4, and pH 8, NaCl concentration of 0.0429 M, in the absence or presence of 1.5 µM Aβ40 seeds, at 37 °C without agitation.
